# Spatio-temporal evolution of water-related ecosystem services: Taihu Basin, China

**DOI:** 10.7717/peerj.5041

**Published:** 2018-06-22

**Authors:** Junyu Chen, Tao Cui, Huimin Wang, Gang Liu, Mat Gilfedder, Yang Bai

**Affiliations:** 1 State Key Laboratory of Hydrology Water Resource and Hydraulic Engineering, Hohai University, Nanjing, China; 2 Land and Water, Commonwealth Scientific and Industrial Research Organization (CSIRO), Brisbane, QLD, Australia; 3 Institute of Management Science, Hohai University, Nanjing, China; 4 Coastal Development and Protection Coordination Innovation Center, Nanjing, China; 5 Center for Integrative Conservation, Xishuangbanna Tropical Botanical Garden, Chinese Academy of Sciences, Xishuangbanna, China

**Keywords:** LULC, Climate change, Urbanization, Water yield, Nutrient retention, Soil retention, InVEST model

## Abstract

Water-related ecosystem services (WESs) arise from the interaction between water ecosystems and their surrounding terrestrial ecosystems. They are critical for human well-being as well as for the whole ecological circle. An urgent service-oriented reform for the utilization and supervision of WESs can assist in avoiding ecological risks and achieving a more sustainable development in the Taihu Basin, China (THB). Spatially distributed models allow the multiple impacts of land use/land cover conversion and climate variation on WESs to be estimated and visualized efficiently, and such models can form a useful component in the toolbox for integrated water ecosystem management. The Integrated Valuation of Ecosystem Services and Tradeoffs model is used here to evaluate and visualize the spatio-temporal evolution of WESs in the THB from 2000 to 2010. Results indicate that water retention service experienced a decline from 2000 to 2005 with a recovery after 2005, while there was ongoing water scarcity in urban areas. Both the water purification service and the soil retention service underwent a slight decrease over the study period. Nutrients export mainly came from developed land and cultivated land, with the hilly areas in the south of the THB forming the primary area for soil loss. The quantity and distribution of WESs were impacted significantly by the shrinkage of cultivated land and the expansion of developed land. These findings will lay a foundation for a service-oriented management of WESs in the THB and support evidence-based decision making.

## Introduction

Water-related ecosystem services (WESs) such as water retention, nutrient retention, soil retention, climate regulation, recreation and biodiversity are the products of the interactions between water ecosystems and their surrounding terrestrial ecosystems, and make contributions to the natural environment and to human well-being (Ouyang et al., 2004; Sample, Baber & Badger, 2016; Hackbart, De Lima & Dos Santos, 2017). The ecosystem service (ES) concept is increasingly accepted and applied in environmental management in order to improve the complex human-environmental systems (De Groot et al., 2010; Müller & Burkhard, 2012; Goldstein et al., 2012; Costanza et al., 2017). Evaluating and mapping of WESs can reveal values and their evolution at different scales, which can help integrate WESs into management practice (Burkhard et al., 2012; Daily & Matson, 2008). WESs have been widely studied and applied (Guo, Xiao & Li, 2000; Grizzetti et al., 2016; Zheng et al., 2016). Recently, instead of focusing on a specific type of ES or on only a few small catchments, studies on ESs typically provide a comprehensive assessment of different types of ESs and their interactions (Bartkowski, 2017; Alamgir et al., 2016; Jopke et al., 2015) and/or cover a large area comprising of multiple sub-bioregions (Mei et al., 2017). Distributed models, such as the SWAT model and the Integrated Valuation of Ecosystem Services and Tradeoffs (InVEST) model, have been used to quantify and map WESs at basin scale (Schmalz et al., 2016; Bai et al., 2011).

The Taihu Basin (THB) has undoubtedly become one of the most prominent sites in terms of tension between insufficient supply and increasing demand of WESs in China, although the abundant WESs had continuously supported the fast growing socio-economic development in this area, particularly from the 1980s. However, climate change and intensive human activities have generated enormous changes in WESs across the whole basin (Hao & Stone, 2010; Xu et al., 2016). Water resources were over-stretched for both industrial and domestic usage, with a local supply of water resources of 145.3 × 10^8^ m^3^ to meet a total demand of 341.4 × 10^8^ m^3^ (domestic: 31.1 × 10^8^ m^3^; industrial and irrigation: 308 × 10^8^ m^3^; ecological restoration: 2.3 × 10^8^ m^3^) (Taihu Basin Authority, 2016). In 2015, less than 30% of water bodies met minimum quality drinking water standards because of pollution (Taihu Basin Authority, 2016). Water-related issues including water scarcity (Zhu, 2003), water pollution (Guo, 2007; Yang & Liu, 2010) and soil loss (Bu et al., 2002) directly influenced the environment, as well as the residents’ living conditions.

Re-establishing a balance between socio-economic development and environmental protection has become the core of the current management plan for the THB. Effective management strategies need to be based on an understanding of the recent spatial/temporal evolution of the WESs. Previous studies have attempted to evaluate the ESs/WESs of the THB at basin scale (Wang et al., 2011; Yan et al., 2015) and sub-ecosystem scale (Xu, Gao & Huang, 2010; Yang et al., 2011; Du et al., 2012). While these studies have advanced our understanding of the WESs in the THB, they typically provide only a total estimation of a specific type of WESs across a region using market-value methods. The results usually do not reflect the realized values of the WESs as they are easily affected by the determination of prices, the selection of indicators, and the researcher’s ecological values. Furthermore, the static total values presented in these existing studies cannot reveal the spatio-temporal distribution of services required for decision-making. Grid-based distributed models have also been applied to study the spatio-temporal evolution of the ESs degradation risks in the THB (Xu et al., 2016), showing that rapid land-use change has posed a great degradation risk of ESs in the THB in 1985–2020. Therefore, a comprehensive evaluation and visualization of the spatio-temporal evolution of WESs would be a useful exercise, in order to identify the major driving factors affecting water ecosystem management in the THB.

The increased demand for spatially-explicit ESs evaluation has led to the development of a wide range of spatially-distributed models which can be used to evaluate ESs at different scales (Arnold et al., 2012; Sherrouse & Semmens, 2012; Villa et al., 2014). The InVEST model, developed by the Natural Capital Project (2016), can be applied to evaluate and map a full suite of ESs to inform decision-makers. It quantifies ESs through integrating land use/land cover (LULC) with various biophysical functions (Vigerstol & Aukema, 2011; Hoyer & Chang, 2014). Outputs of the InVEST model help clarify the generation mechanisms of ESs and can be used to judge the positive and negative impacts of different polices on the ecosystem (Daily et al., 2009; Nelson et al., 2009). To date, it has been successfully applied to a number of sites across the world at multiple scales to support ecosystem management and economic development planning, such as Hawaii (Daily et al., 2009), Arizona (Bagstad, Semmens & Winthrop, 2013), Minnesota (Polasky et al., 2011), Oregon (Nelson et al., 2009), UK (Redhead et al., 2017), Llobregat basin in Spain (Terrado et al., 2014), Ghana and Côte d’Ivoire (Leh et al., 2013), Colombia (Goldman et al., 2010) and China (Chen et al., 2011; Zhang et al., 2012; Jiang et al., 2017).

The InVEST model has previously been applied in the THB to estimate water yield in the West Tiaoxi Basin (a sub-basin of the THB) (Zhang et al., 2012) and to map the distribution of (and interaction between) multiple ESs of the Jiangsu section of the THB (Ai et al., 2015). However, both of these studies are based on a single year’s data and are conducted at a sub-basin scale. A comprehensive evaluation of multiple WESs covering the entire THB for multiple years will better help to support an evidence-based integrated water ecosystem management for the THB. This study aims to evaluate three types of WESs including water retention service, water purification service and soil retention service by applying the InVEST model across the entire THB. Three aspects are of particular interest to this study: (1) the spatio-temporal evolution of the three specific types of WESs in the THB for the years 2000, 2005 and 2010; (2) the major driving mechanisms of the spatio-temporal variation; and (3) the implications of WESs for service-oriented solutions to water ecosystem management.

## Materials and Methods

### Study area

**Figure 1:**
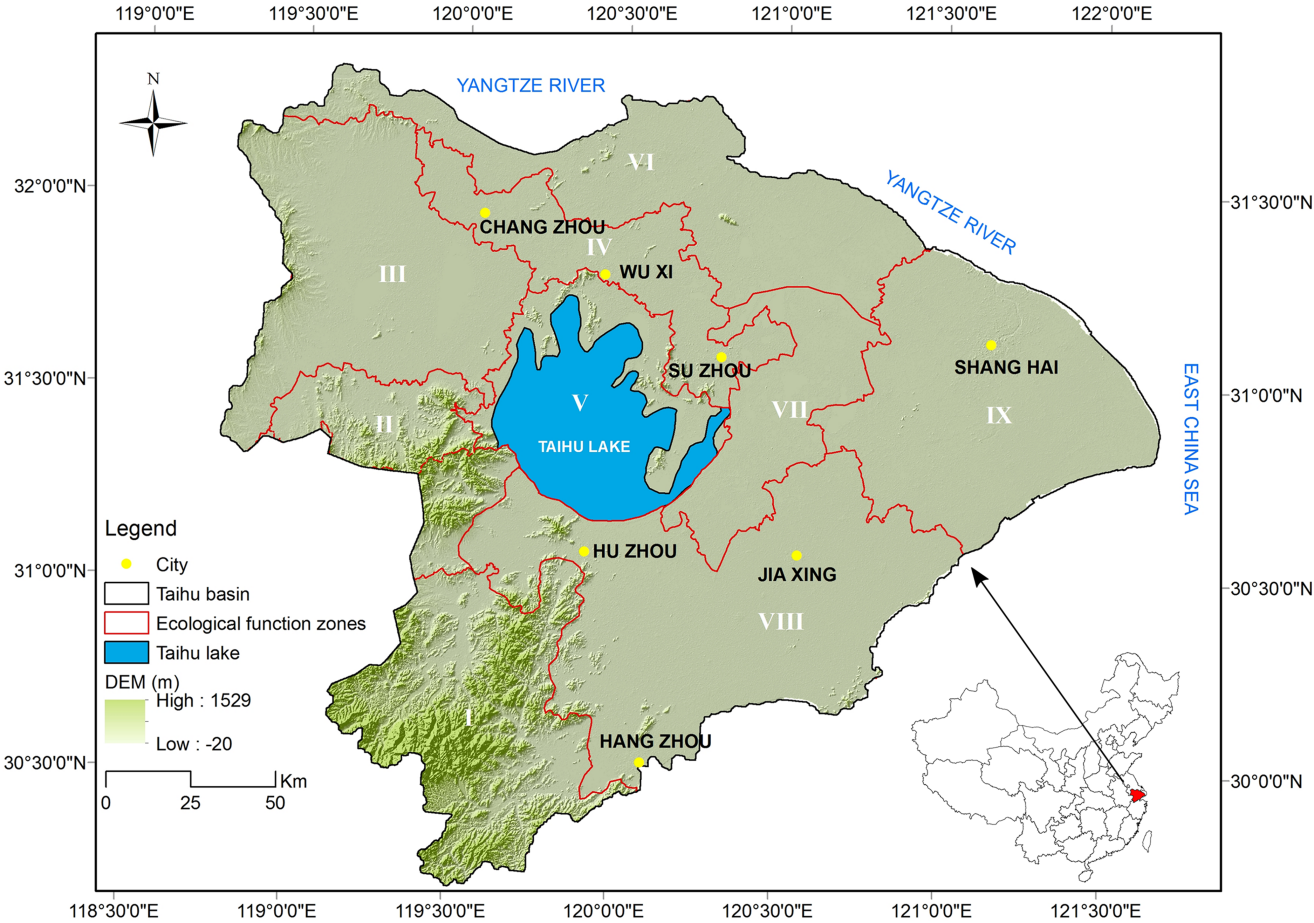
Location of the Taihu Basin and its elevation; nine sub-regions (I–IX) are divided according to the Chinese national ecological function zoning (2013) for regional analysis.

The THB (Fig. 1) is the core part of the Yangtze River Delta covering an area of 36,895 km^2^. It lies within the subtropical monsoon climate zone, with a long-term annual rainfall of 1,177 mm and an annual average temperature of 16.2 °C. The THB has a complex surface-water system with a river density of 3.3 km/km^2^ and six lakes (above 50 km^2^). Encompassing several big cities including Shanghai, Suzhou and Wuxi, the THB is one of the most urbanized zones in China with a population of 59.97 million, and in 2015 it produced ~9.9% of China’s GDP from only 0.4% of the land area (Taihu Basin Authority, 2016).

### Land use/land cover change

**Figure 2:**
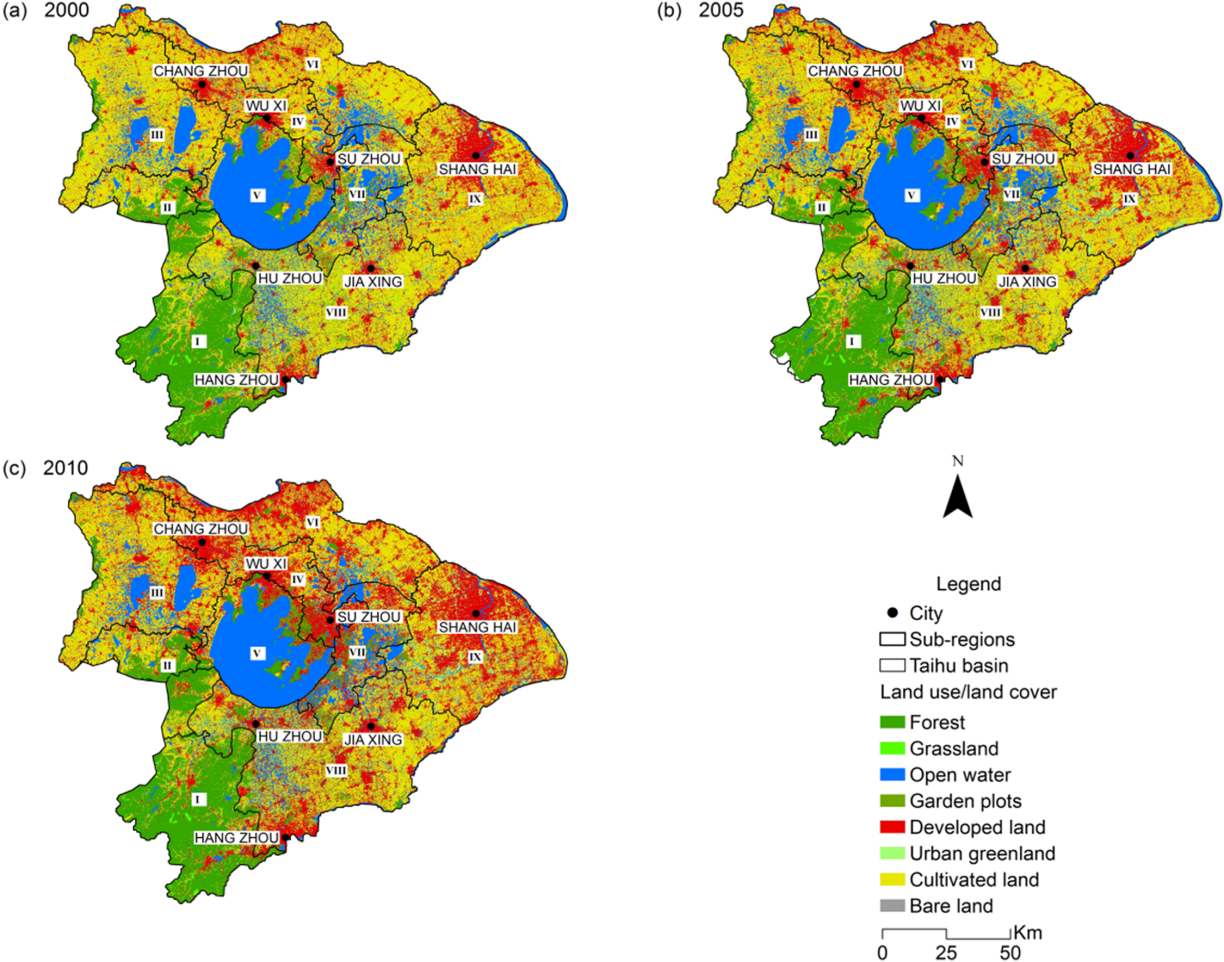
Comparison of the land use/land cover of the Taihu Basin in 2000 (A), 2005 (B), 2010 (C).

Rapid and intensive urbanization led to enormous transformation of LULC from 2000 to 2010 (Fig. 2; Table 1). The total area of developed land expanded significantly from 5,661 to 10,082 km^2^ over the 2000–2010 period, in order to match the increasing population and industries, and it was mostly converted from cultivated land (3,952 km^2^) and open water (332 km^2^). There was a massive reduction in cultivated land over this period, shrinking from 18,613 km^2^ in 2000, to 16,964 km^2^ in 2005 and to 14,228 km^2^ in 2010. While 21.23% of cultivated land was converted into developed land, 1.34% and 1.09% of it was also changed into urban greenland (urban greenland refers to natural and artificial vegetated areas, mainly including parks and residential green space) and forests respectively. Grassland experienced a 53.64% sharp decrease, with 90% of this reduction becoming cultivated land. Land used for garden plots (garden plots belongs to the secondary classification of cultivated land, mainly including orchards and tea plantations) and bare land fluctuated slightly through the decade. The transformation metrics also revealed that the conversions that occurred on developed land and cultivated land from 2005 to 2010 were more obvious than for the period of 2000–2005, which is consistent with the rate of urbanization in the THB. In contrast, the changing intensity of the rest of the LULCs were more significant in the first period (2000–2005). Suburbs and affiliated counties of the core cities including Shanghai, Suzhou, Wuxi and Changzhou experienced dramatic land use transformation and the most notable conversion was from cultivated land to developed land.

**Table 1:** Land use/land cover transition matrix of the Taihu Basin from 2000 to 2010 (unit: km^2^).

		Cultivated land	Developed land	Forest	Garden plots	Grassland	Bare land	Urban greenland	Open water	Total
		2010 LULC								
2000 LULC	Cultivated land	13,693	3,952	203	49	0	1	250	465	18,613
	Developed land	18	5,627	3	0	0	0	4	9	5,661
	Forest	0	39	4,880	0	0	0	2	5	4,926
	Garden plots	13	4	20	811	0	0	0	1	849
	Grassland	27	121	11	2	140	0	0	1	302
	Bare land	1	0	0	0	0	0	0	3	4
	Urban greenland	5	7	0	0	0	0	193	4	209
	Open water	471	332	2	21	0	0	11	5,564	6,401
	Total	14,228	10,082	5,119	883	140	1	460	6,052	36,965

		2005 LULC								
2000 LULC	Cultivated land	14,729	2,515	360	141	30	1	170	668	18,613
	Developed land	1,021	4,314	55	46	7	0	50	167	5,661
	Forest	265	65	4,524	30	14	1	1	25	4,926
	Garden plots	106	61	33	614	1	0	0	34	849
	Grassland	76	72	27	4	114	0	0	9	302
	Bare land	1	0	1	0	0	2	0	0	4
	Urban greenland	23	42	1	0	0	0	136	7	209
	Open water	744	293	34	40	5	0	14	5,271	6,401
	Total	16,964	7,363	5,034	875	173	4	372	6,180	36,965

		2010 LULC								
2005 LULC	Cultivated land	12,235	3,455	278	132	18	1	115	730	16,964
	Developed land	958	6,004	74	63	7	0	62	195	7,363
	Forest	198	152	4,607	22	12	1	6	35	5,034
	Garden plots	60	115	40	610	1	0	1	47	875
	Grassland	27	25	17	1	98	0	0	5	173
	Bare land	1	0	1	0	0	2	0	0	4
	Urban greenland	28	74	1	0	0	0	256	14	372
	Open water	722	338	29	53	4	0	17	5,017	6,180
	Total	14,228	10,163	5,047	882	140	5	457	6,043	36,965

### Climatic condition

The THB experienced climatic variability over the 2000–2010 period. Figure S1A shows the spatial distribution of the 30-year average precipitation (1980–2010) in the THB. The 30-year average was above 1,610 mm/year in the south–west of the study area; and it decreased gradually to below 1,120 mm/year in the north–east. The annual average precipitation across the THB was 1,280 mm in 2000, 967 mm in 2003, and then 1,222 mm in 2010 (Fig. S1B). While the east had the highest annual average temperature of above 16.5 °C, the southwest was below 10 °C (Fig. S1C). The annual average temperature across the study area fluctuated through the whole period, however, an overall warming tendency could be found (Fig. S1D) (Taihu Basin Authority, 2016).

### Models

Considering both the local government’s concern and residents’ benefits, this study selects the Water Yield model (for water retention service), Nutrient Delivery Ratio model (for water purification service) and Sediment Delivery Ratio model (for soil retention service) from the InVEST model 3.2.1 (Natural Capital Project, 2016) to evaluate the spatial-temporal change of WESs in the THB. Details of these models with their governing equations are provided in the supplementary material (File S1). Only the definition of the estimated WESs and their evaluation principles are described very briefly here.

**Water retention service:** water retention service is defined as the ability of ecosystems to intercept or store water resources from rainfall, which can be quantitatively described as annual water yield. Annual water yield is estimated based on annual average precipitation and the Budyko curve (Budyko, 1974).

**Water purification service:** water purification service in this study refers to the ecosystem’s capacity to absorb nitrogen and phosphorus from the water flow. Sources of nutrient across the landscape are determined based on the land use map and the associated loading rates for different types of lands.

**Soil retention service:** soil retention service accounts for the retention of rainfall-eroded soil by vegetation, for the protection of soil resources and water quality. The InVEST sediment delivery model was used to map the overland sediment generation and delivery to streams.

### Data requirement and preparation

As described above, the InVEST model requires multiple gridded data sets together with specific biophysical data as inputs. The collected spatial data for THB and other relevant crucial data are listed in Table S1. The table summarizes each dataset by their sources, a short introduction and the associated models. Table S2 then lists the key parameters required by the InVEST model, and Table S3 is the required biophysical table.

## Results

### Evolution of water-related ecosystem services

**Figure 3:**
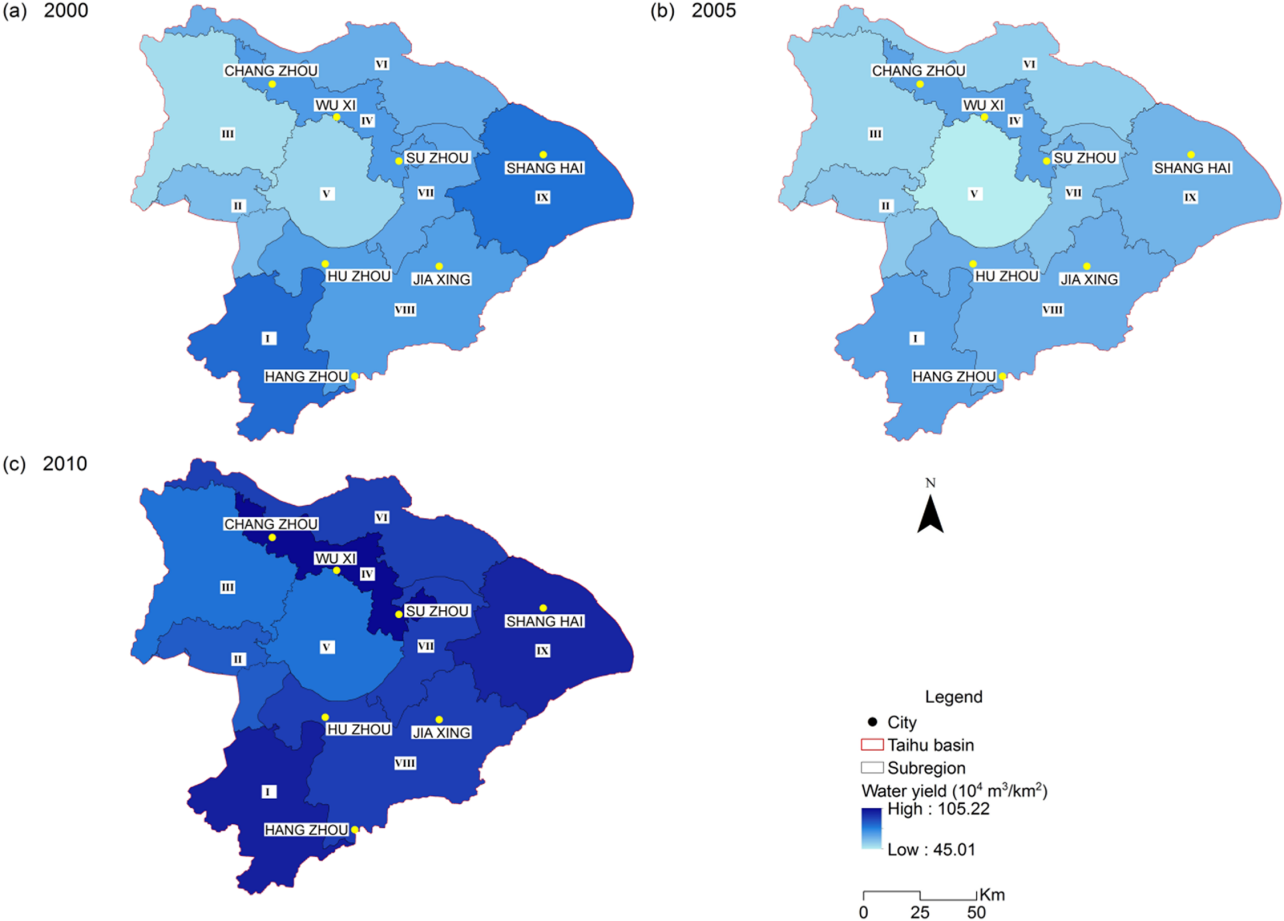
Spatial distribution of water yield of the Taihu Basin in 2000 (A), 2005 (B), 2010 (C).

**Figure 4:**
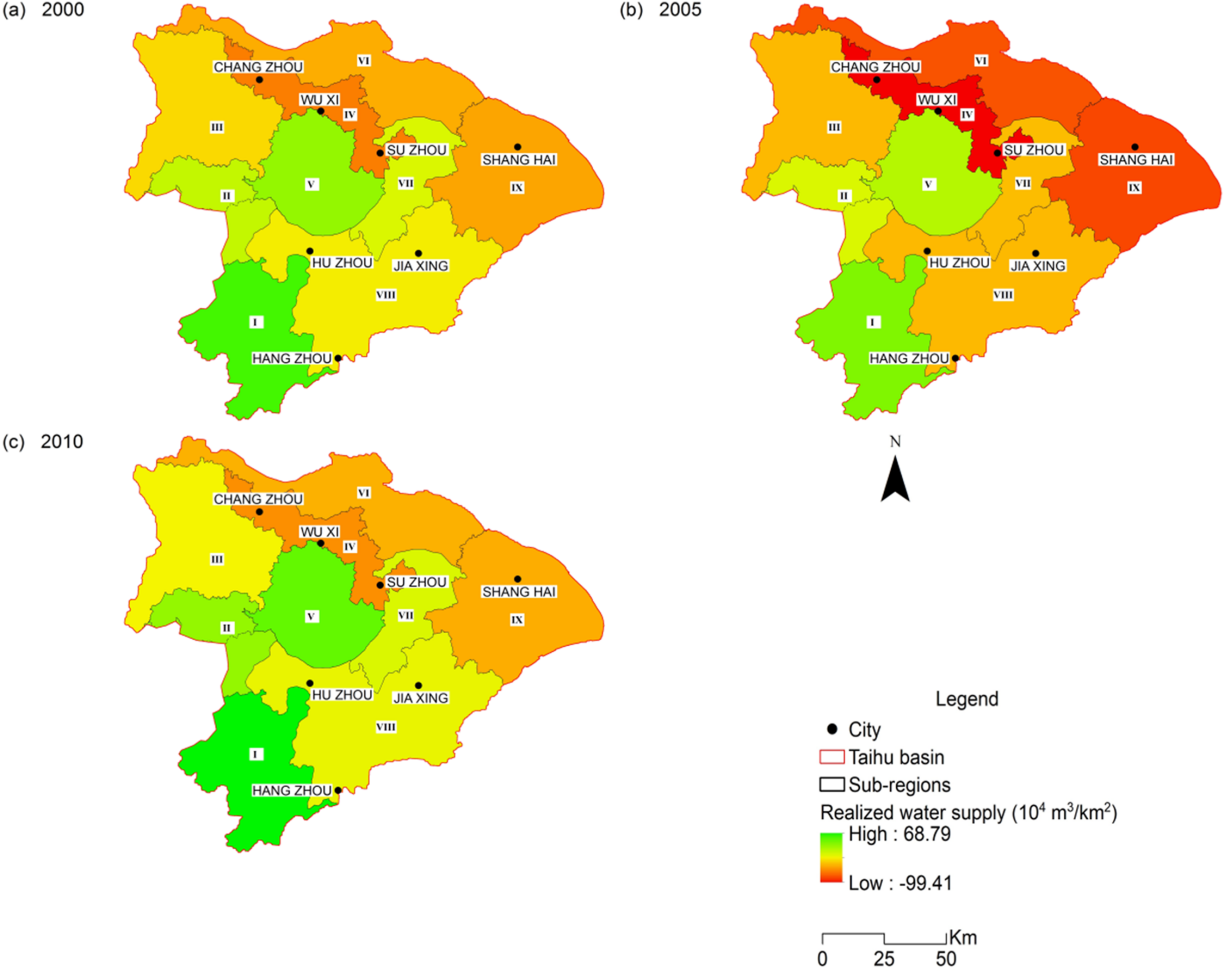
Spatial distribution of water scarcity of the Taihu Basin in 2000 (A), 2005 (B), 2010 (C).

Water-related ecosystem services in the THB showed various spatial patterns and evolutions during the modelled period. Regarding the water retention services, the total volume of yielding water decreased from 241.76 × 10^8^ m^3^ in 2000 to 213.19 × 10^8^ m^3^ in 2005, and rebounded to 336.83 × 10^8^ m^3^ in 2010 (Table 2). Sub-region I showed strong water-yield capability (Fig. 3) as a result of rich rainfall and high vegetation coverage. Sub-region IV and IX also produced larger amounts of water resources compared to the rest, mainly due to the expansion of developed land with low evapotranspiration. Water yield from sub-regions III, IV and V increased by 60%, 56% and 53% from 2000 to 2010, respectively. Despite the increase in total water yield over the modelled period, water scarcity had been continuously present in most of the heavily populated sub-regions (Fig. 4). Due to the high density of population and large industrial scale, water demand in sub-regions IV, VI and IX (where developed cities like Shanghai, Suzhou and Wuxi are located) is far more than the available local supply.

**Table 2:** Total amount of water-related ecosystem services in the Taihu Basin in 2000, 2005 and 2010.

Water-related ecosystem services	2000	2005	2010
Water yield (10^8^ m^3^)	241.76	213.19	336.83
Nitrogen load (ton)	134,734	129,180	121,888
Nitrogen load (ton/km^2^)	3.65	3.50	3.30
Nitrogen retention (ton)	105,051	100,749	94,770
Nitrogen retention (ton/km^2^)	2.85	2.73	2.57
Nitrogen export (ton)	29,683	28,432	27,119
Retained ratio of nitrogen	77.97%	77.99%	77.75%
Phosphorus load (ton)	22,611	22,377	22,236
Phosphorus load (ton/km^2^)	0.61	0.61	0.60
Phosphorus retention (ton)	17,630	17,445	17,259
Phosphorus retention (ton/km^2^)	0.48	0.47	0.46
Phosphorus export (ton)	4,981	4,932	4,978
Retained ratio of phosphorus	77.96%	77.96%	77.62%
Soil loss (10^8^ ton)	5.67	4.05	5.38
Soil retention (10^8^ ton)	5.44	3.90	5.17
Soil export (10^8^ ton)	0.23	0.15	0.21

**Figure 5:**
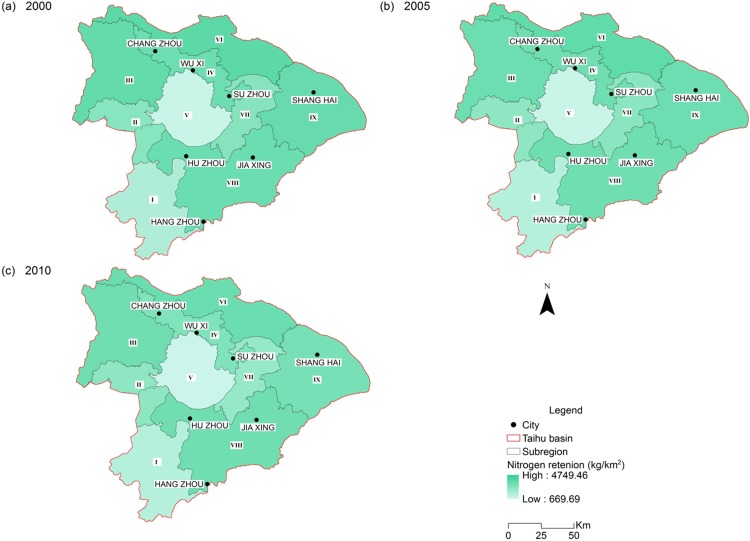
Spatial distribution of nitrogen retention of the Taihu Basin in 2000 (A), 2005 (B), 2010 (C).

**Figure 6:**
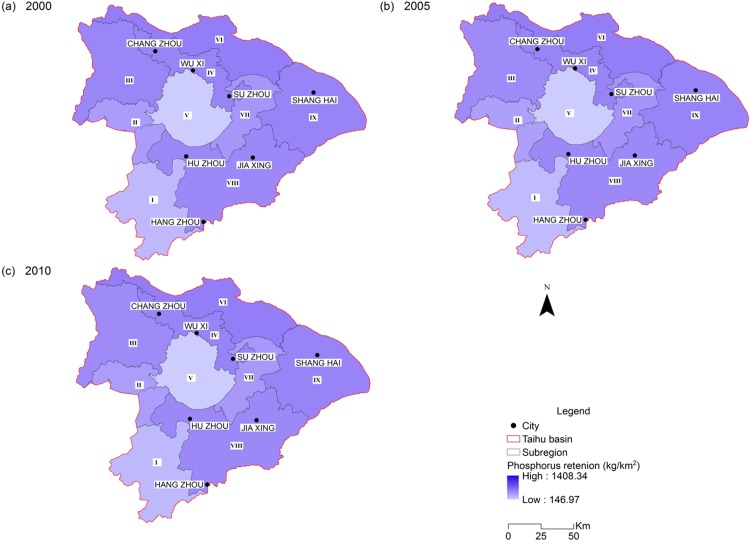
Spatial distribution of phosphorus retention of the Taihu Basin in 2000 (A), 2005 (B), 2010 (C).

Estimates of nitrogen and phosphorus load reduced by 9.53 % and 1.66 % respectively during the study period. This was reflected by a 9.79 % decline of retained nitrogen and a 2.10 % decline of retained phosphorus. The nitrogen exported to streams also experienced a slight decrease from 29,683 ton/year in 2000 to 27,119 ton/year in 2010, as well as the exported phosphorus from 4,981 ton/year in 2000 to 4,978 ton/year in 2010 (Table 2). This is generally consistent with the improvement of the water quality during the decade, as the nitrogen and phosphorus are the dominant factors for the THB water pollution. The spatial pattern of retention service for nitrogen and phosphorus are very similar due to their similar processing mechanism in the model, which is consistent with the findings of Bai et al. (2011) for the Baiyangdian watershed, China. Nutrient retention amount was high in sub-regions III, VI and IX (Figs. 5 and 6; Table 3). Besides the retention capacity of the ecosystem in these sub-regions, this may be partly because that nutrient loads are usually high due to intensive agricultural activity and large population in the plain areas.

**Table 3:** Nutrient load, retention and export at sub-region level in 2000, 2005 and 2010.

	Nitrogen load (ton)	Nitrogen retention (ton)	Nitrogen export (ton)	Phosphorus load (ton)	Phosphorus retention (ton)	Phosphorus export (ton)
	2000					
Sub-region I	8,938	7,167	1,771	2,431	1,928	503
Sub-region II	6,532	4,955	1,576	1,899	1,435	465
Sub-region III	20,288	15,436	4,852	6,039	4,594	1,445
Sub-region IV	9,701	7,415	2,286	2,847	2,176	671
Sub-region V	3,115	2,447	667	902	707	195
Sub-region VI	19,788	15,153	4,634	5,869	4,495	1,374
Sub-region VII	7,128	5,601	1,527	2,115	1,662	453
Sub-region VIII	31,916	25,005	6,911	9,454	7,405	2,049
Sub-region IX	25,020	19,325	5,695	7,362	5,686	1,676

	2005					
Sub-region I	7,992	6,759	1,233	2,145	1,928	218
Sub-region II	6,628	5,397	1,232	1,922	1,716	206
Sub-region III	21,294	17,354	3,940	6,320	5,677	643
Sub-region IV	9,229	7,437	1,792	2,686	2,349	337
Sub-region V	3,061	2,517	544	881	775	106
Sub-region VI	17,919	14,587	3,332	5,286	4,710	577
Sub-region VII	6,613	5,468	1,145	1,948	1,737	211
Sub-region VIII	31,955	26,667	5,288	9,431	8,537	894
Sub-region IX	20,799	17,028	3,771	6,074	5,403	672

	2010					
Sub-region I	7,862	6,578	1,285	2,096	1,861	235
Sub-region II	6,264	5,084	1,180	1,806	1,603	203
Sub-region III	19,374	15,732	3,642	5,723	5,105	618
Sub-region IV	8,219	6,522	1,698	2,353	2,002	351
Sub-region V	2,666	2,155	512	752	641	110
Sub-region VI	17,399	14,073	3,326	5,093	4,483	610
Sub-region VII	5,684	4,644	1,040	1,654	1,446	209
Sub-region VIII	29,267	24,235	5,031	8,589	7,702	886
Sub-region IX	18,751	15,217	3,534	5,400	4,722	679

**Figure 7:**
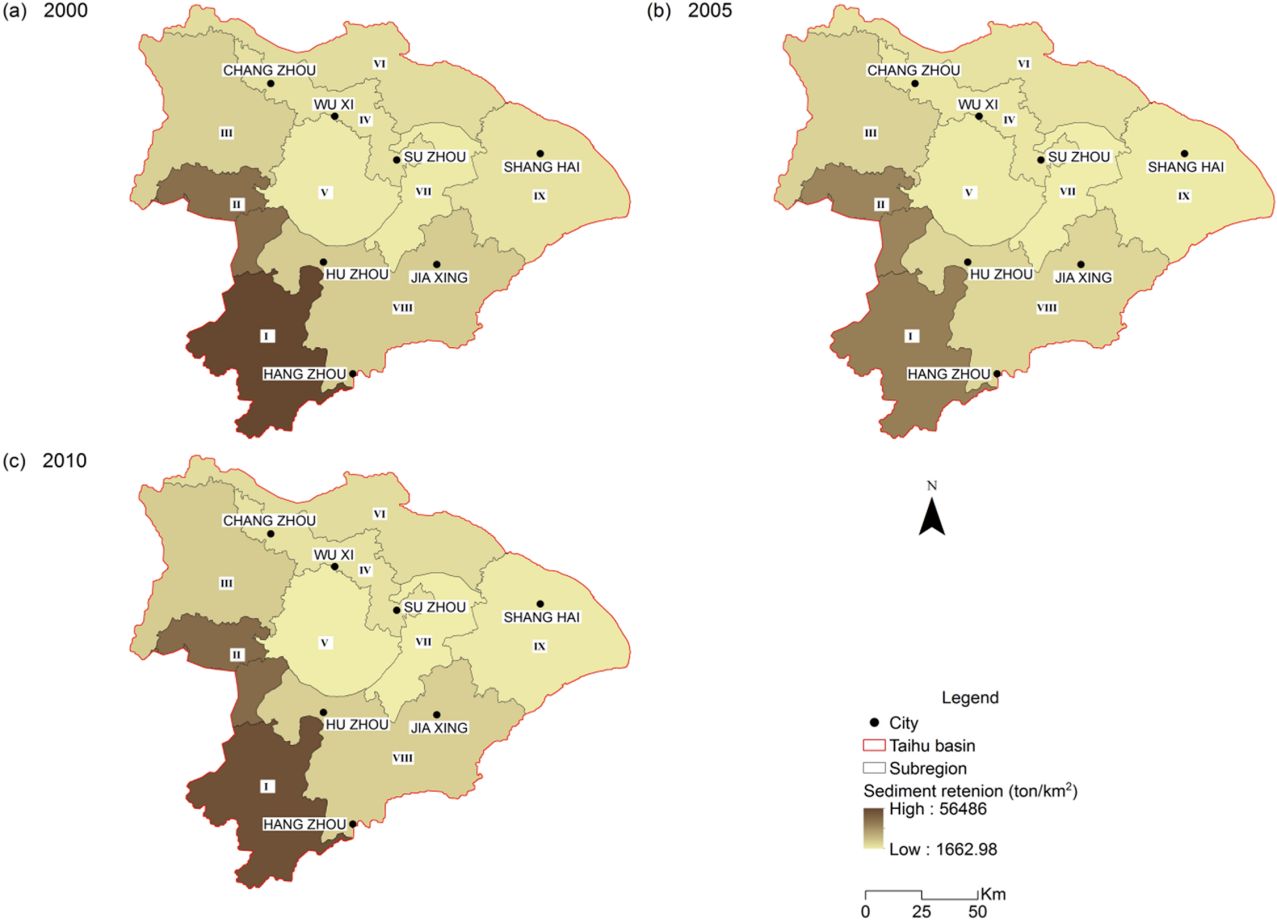
Spatial distribution of sediment retention of the Taihu Basin in 2000 (A), 2005 (B), 2010 (C).

Total soil retention was estimated to decline to 3.90 × 10^8^ ton/year in 2005 from 5.44 × 10^8^ ton/year in 2000, but then increased to 5.17 × 10^8^ ton/year in 2010. Overall, the total soil loss across the study area was reduced by (5.11%), with a declining soil export by (8.70%) as a result of improving soil retention capacity. Although the hilly areas (sub-region I, II and III) were mostly covered with natural vegetation, which is typically more efficient at retaining soil, these areas still contribute the most sediments exporting to streams, as a result of mountainous landscape (Fig. 1) and high precipitation (Fig. S1). Over time, those northern and eastern plain areas only showed slight changes, while the hilly areas experienced soil retention services fluctuations (Table 2; Fig. 7).

The retention results must be interpreted with caution because the retention service is a variable that is largely controlled by the nutrient and sediment loading and the retention capacity of the ecosystem in a specific area. For example, the nutrient retention amount cannot be directly used as an indicator for retention efficiency and retention capacity. The leafy sub-region I may have a higher retention capacity than other sub-regions. However, the nutrient loading in this region is much lower than other sub-regions with agricultural lands. The lower nutrient loading makes it as a region with low retention service (Figs. 5 and 6).

### Correlation among water-related ecosystem services

Different types of ESs are functions of LULC and various biophysical processes, and are typically interdependent, and thus can be represented as either a conflict or a synergy at spatio-temporal scales. A *Pearson Correlation Analysis* was conducted to investigate the interactions among different types of WESs based on the results of every sub-region (Table 4).

**Table 4:** Correlation matrix among estimated water-related ecosystem services and drive factors.

(A)	Developed land ratio	Natural ecology land ratio	Cultivated land ratio	Precipitation	Water yield	Water scarcity			
Developed land ratio	1								
Natural ecology land ratio	−0.553**	1							
Cultivated land ratio	0.478*	−0.406*	1						
Precipitation	−0.115	0.394*	−0.048	1					
Water yield	0.390*	0.172	0.100	0.835**	1				
Water scarcity	0.561*	−0.299	−0.168	−0.514*	−0.314	1			
Nitrogen retention	0.565**	−0.503**	0.879**	−0.377	−0.107	0.481*			
Phosphorus retention	0.797**	−0.546**	0.739**	−0.329	0.095	0.704**			
Nitrogen load	0.553**	−0.504**	0.888**	−0.372	−0.115	0.480*			
Phosphorus load	0.799**	−0.585**	0.750**	−0.314	0.097	0.699**			
Nitrogen export	0.563**	−0.477*	0.879**	−0.322	−0.068	0.449			
Phosphorus export	0.798**	−0.451*	0.744**	−0.201	0.195	0.633**			
Sediment retention	−0.414*	0.623**	0.216	0.343	0.156	−0.432			
Sediment loss	−0.414*	0.623**	0.216	0.343	0.156	−0.432			
Sediment export	−0.462*	0.628**	0.201	0.305	0.092	−0.422			

(B)	Nitrogen retention	Phosphorus retention	Nitrogen load	Phosphorus load	Nitrogen export	Phosphorus export	Sediment retention	Sediment loss	Sediment export
Nitrogen retention	1								
Phosphorus retention	0.907**	1							
Nitrogen load	0.995**	0.896**	1						
Phosphorus load	0.900**	0.987**	0.899**	1					
Nitrogen export	0.951**	0.858**	0.968**	0.886**	1				
Phosphorus export	0.837**	0.919**	0.846**	0.951**	0.894**	1			
Sediment retention	−0.433*	−0.445*	0.073	−0.125	0.125	−0.018	1		
Sediment loss	0.048	−0.124	0.073	−0.125	0.125	−0.018	1.000**	1	
Sediment export	0.042	−0.147	0.071	−0.144	0.13	−0.035	0.991**	0.991**	1

**Notes:**

* indicates significant level *p* < 0.05;

** indicates significant level *p* < 0.01.

The highest correlation was observed between the nitrogen retention and phosphorus retention (*r*^2^ = 0.907, *p* < 0.01) due to their similar processing mechanism. This is consistent with the study in the Baiyangdian basin, China (Bai et al., 2013). The results show that soil retention was negatively related to nutrient retention with a correlation coefficient of −0.433 (*p* < 0.05) and −0.445 (*p* < 0.05) for nitrogen and phosphorus, respectively. The counter-intuitive result is a result of spatial variation of the relevant WESs. The two services are typically present in different parts of the watershed. Soil retention more often takes place within hilly areas, while the nutrient retention tends to occur on developed land and cultivated land within the plain areas. Thus, when major controlling variables, such land use, are very different among sub-regions, correlation analysis is not an efficient tool to reveal relationships between different WESs.

### Driving mechanisms for water-related ecosystem services

Among various factors driving the evolution of WESs, LULC conversion and climate variation play a more profound role in the structure and function of water ecosystems in the THB. The annual precipitation of THB fluctuated to a low level in 2005 compared to that in 2000, but then experienced a steady increase until 2010 (Fig. S1). As a result, the total water yield represented by the discrepancy between precipitation and evapotranspiration turned out to decrease at first and rebound after year 2005, while the year 2010 saw a higher level (Fig. 3).

The shrinkage of both cultivated land (23.56%) and grassland (53.64%) together with the significant expansion of developed land (79.53%) during the evaluated period considerably reduced total evapotranspiration in THB. These LULC conversions resulted in higher water yield in urban areas (Fig. 3). The increasing water yield due to LULC conversion can be observed in sub-region IX (Fig. 3). Although the expansion of developed land leads to higher water yield, developed land ratio presented a positive correlation with the water scarcity (*r*^2^ = 0.561, *p* < 0.05). This is mainly because of the higher water demand due to the growing population and industry. Much of the extra water yield due to the loss of water retention service becomes urban runoff (Lusk & Toor, 2016), which could be collected as a new water source.

Although a large amount of nutrient discharge comes from waste water, the main source of nutrient pollution in the THB is from fertilizer application for agricultural production (Li, Zhang & Ren, 2008). With the reduction of cultivated land at basin scale, the total load of nitrogen and phosphorus decreased slightly. Spatially, nutrient retention was concentrated in the plain areas. This is because cultivated land and developed land (with relatively high load of nitrogen and phosphorus), are mainly distributed in the plain areas where the slow flow velocity promotes the retention capacity of vegetation. However, rapid increases of developed land resulting from the expansion of the large cities significantly weakened the nutrient retention services.

Implemented by the National Grain for Green Program (Zhou et al., 2009), the conversion from cultivated land to forests and garden land in hilly areas effectively reduces the risk of soil erosion (Kong et al., 2018). The program was initiated across China in 1999. Specifically, the program was implemented in the THB to improve water quality, strengthen soil conservation and increase water provisioning. This program resulted in a large decrease of agricultural land in the THB, especially in the hilly areas (mainly in sub-region I). This conversion resulted in not only a decrease of total soil loss over the study period, but also an increase of soil retention due to increased vegetation cover. This is consistent with the positive impacts of natural ecological land ratio on soil retention (*r*^2^ = 0.623, *p* < 0.01).

## Discussion

### Potential water-related ecological risks

Our evaluation reveals the changes occurred to the three estimated WESs in the THB between years of 2000, 2005 and 2010. Based on the evaluation, we suggest that some potential ecological risks may pose threats to the future development of the THB.

For water provisioning, Fig. 4 shows the imbalance of water demand and supply. The local water resources cannot support the sustainable development of the THB, particularly in sub-regions where large cities exist. This fled to a project to divert water from the Yangtze River to Taihu Basin to satisfy this growing demand (Zhai, Hu & Zhu, 2010).

On the other hand, the extra water yield, such as sub-regions IV and VII, from the expanding developed land may escalate the risks of flooding in the THB. LULC changes caused by urbanization will continue in the next decade in the THB. According to the governing equations of the InVEST model, developed land with high precipitation and low evapotranspiration should produce a large water yield. Meanwhile, impermeable materials now cover most of the THB’s cities, which severely limits rainfall infiltration in these areas. As a result, the extra water yield from the urban areas where developed lands dominate mainly becomes urban runoff. Rainwater harvesting to convert the increased water yield into useful local water resource could help to reduce water scarcity and flooding risk.

It is notable that the decline of cultivated land slightly reduced the nutrient export to streams in the THB. However, the proportion of developed land in the THB will inevitably increase with the development. This may lead to increasing nutrient discharge from household and industry waste in the future. Additionally, sub-regions (e.g., sub-region VIII) that rely on crop production may attempt to maintain high outputs through the higher utilization of agrochemicals with shrinking cultivated land (Liao, Peng & Luo, 2005). Therefore, the load of nitrogen and phosphorus is likely to rebound later. Combined with modelled reduced capacity for nutrient retention, it seems likely that nitrogen and phosphorus pollution will continue in the future.

For the soil conservation, this study indicates that overall soil loss in the THB stayed at a low level, while mass loss mainly occurred in the hilly areas located in the southwest (as concluded by Zhang & Meng (2009). Despite this, the potential for soil loss within urban areas cannot be ignored. During 2005–2010, there was a lot of real estate development. Few of these developments had any soil conservation plan, resulting in the loose soil being transported away by heavy rain. Our modelling results also shows an increasing trend for soil loss in the eastern and northern developed cities from 2005 to 2010 (Fig. 6). Therefore, this study argues that the risk of soil loss in the THB remains.

### Service-oriented management of water ecosystems

The modelling results and findings can be used to inform service-oriented management. This study proposes the following service-oriented actions to improve the integrated water resources management in the THB:

1. Areas with a specific type of superior WESs (and the surrounding buffer zones) could be designated as corresponding ecological functional regions to ensure the sustainable provision of WESs. For instance, a water conservation area could be established in sub-region I due to its strong yielding ability. For those areas severely lacking a specific type of required WESs, ecological restoration measures can be implemented, and their effectiveness quantitatively measured by the recovery of WESs. Sub-region IX with serious water shortage could investigate new ways to increase their local water supply, such as rainfall harvesting and waste-water recycling. Inspired by the *Low-impact Development* strategy in the USA and *Water Sensitive Urban Design* in Australia (Eckart, Mcphee & Bolisetti, 2017; Morison & Brown, 2011), China has begun to take measures to transfer cities with serious water scarcity into “sponge cities,” which has achieved remarkable success (Liu, 2016; Liu, Jia & Niu, 2017);
2. Cultivated land is one of the dominant LULCs for the evolution of WESs in the THB, and is especially important for nutrient retention services. Therefore, measures which reduce nutrient loading and enhance nutrient retention would be beneficial. As an example, vegetation isolation zone or nutrient collection drainage may be set up in the sub-region VIII surrounding the cultivated land to prevent the redundant nutrients from exporting to streams;
3. The urbanization ratio (urban percentage of the total population) in the THB was 65% in 2010 and it is expected to reach 82.7% in 2020, which is much higher than the national target (60%) (Li, 2013). As the most significant symbol of urbanization, the expansion of the developed land in the THB has weakened interactions among different ecosystems since constructed pavement and buildings were not purposely designed to extend existing natural ecosystem functions. Thus, the complete cycle of ecological process should be considered during the planning of developed land;
4. The modelling results can also be used to guide monitoring site selection. Additional monitoring sites would increase our understanding of the WESs evolution and provide data to help restore ecosystems. The monitoring data would also help establish an adaptive punishment strategy.

### Limitations and prospects

While this study has provided insights into the spatio-temporal evolution of the WESs of the THB, the following limitations need to be noted: (1) the InVEST model has been continuously improved since its first release in 2008, however, some ecological and water-related physical–chemical processes are still relatively simplistic, which should be considered when interpreting model outputs (Sharp et al., 2016). For instance, water yield is simplified to be the residual between precipitation and evapotranspiration. However, in reality, part of the precipitation will become recharge for groundwater depending on climate and other hydrogeological conditions. (2) Both the NDR model and SDR model are strongly reliant on the flow direction as defined by the DEM. 83% of the area in the THB are plains and has been heavily changed by human activity, such as a high-density drain network, which make it more difficult to obtain flow directions correctly in our study. Therefore, we suggest that the InVEST model is more suitable for research on mountainous areas or natural ecosystems. (3) Due to lack of data availability, some of the input parameter values were sourced from the official user guide of the InVEST model or literature, rather than being customized with local information. Thus, further study to provide locally refined and calibrated parameters will benefit the results. (4) Scale effect is regarded as an important factor influencing the ESs evaluation and management (Xu et al., 2017). Different evaluating scales determine different distribution patterns of hot spot of ESs. For instance, sub-region I was estimated to be the hot spot with most yielding water, however, the city of Shanghai would replace it as the new hot spot if “city” had been selected as our modelled evaluation scale. Furthermore, this promotes the stakeholders on different scales (e.g., basin/city/county, etc.) to implement different policies. In this study, policies are proposed only at basin or sub-region scale. (5) Previous research is mainly concerned with the supply side, while the demand side has not yet received much attention (Li et al., 2016; Wolff et al., 2017). Future research could attempt to evaluate the actual demand of specific WESs, to propose a pricing model based on the supply-demand structure to estimate the actual economic values of different WESs.

## Conclusion

This study investigated the spatio-temporal evolution of WESs in the Taihu Basin using the InVEST model. The overall condition of water ecosystem had been slightly improved from 2000 to 2010 according to the estimated WESs. However, potential ecological risks still exist. Rapid urbanization, which caused unprecedented LULC change, significantly affected both the total amount and the spatial distribution of WESs in the basin. Service-oriented management strategy is suggested to achieve a more sustainable development.

Distributed models based on ecological processes, such as the InVEST model, are powerful tools to analyze and visualize the intertwined interactions between ESs and their driving factors. The results presented in this study provide evidence-based support for the implementation of an integrated water ecosystem management framework in the Taihu Basin.

## Supplemental Information

10.7717/peerj.5041/supp-1 Supplemental Information 1 Water-related ecosystem services evaluation models. Details of the Water Yield model (for water retention service), Nutrient Delivery Ratio model (for water purification service) and Sediment Delivery Ratio model (for soil retention service) from the InVEST model (version 3.2.1).

10.7717/peerj.5041/supp-2 Supplemental Information 2 Spatial distribution of 30-year average annual (a) precipitation and (c) temperature, and temporal evolution of (b) precipitation and (d) temperature from 2000 to 2010 in the Taihu Basin. Four sub-figures indicate the spatio-temporal condition of precipitation and temperature in the Taihu Basin.

10.7717/peerj.5041/supp-3 Supplemental Information 3 Data requirement for the InVEST model (Water yield model = WY; Nutrient delivery ratio model = NDR; Sediment delivery ratio model = SDR). This table lists collected spatial data and other relevant crucial data for the Taihu Basin water-related ecosystem services evaluation. It also summarizes each dataset by their sources, and includes a short introduction and the associated models.

10.7717/peerj.5041/supp-4 Supplemental Information 4 Key parameters used in the current study. This table lists the key parameters required by the InVEST model.

10.7717/peerj.5041/supp-5 Supplemental Information 5 Biophysical table. This is the required biophysical table for the InVEST model.

## Additional Information and Declarations

### Data Availability

The following information was supplied regarding data availability: All data and corresponding sources are in the “Data requirement and preparation” section and Tables S1–S3.
