# Supplementary material for: Spatio-temporal evolution of water-related ecosystem services: Taihu Basin, China
Source: PeerJ. 2018 Jun 22;6:e5041. doi: 10.7717/peerj.5041 (PMC6016528; doi:10.7717/peerj.5041)
Supplement: Supplemental Information 1 — Details of the Water Yield model (for water retention service), Nutrient Delivery Ratio model (for water purification service) and Sediment Delivery Ratio model (for soil retention service) from the InVEST model (version 3.2.1). [file peerj-06-5041-s001.docx]

**Supplemental file 1 (SF 1):**

**Water-related ecosystem services evaluation models**

**Water yield (WY) model:** Water retention service is defined as the ability of ecosystems to retain water resources from rainfall, which can be quantitatively described as annual water yield. The annual water yield for pixel $i$ on $LULC_{j}$, $Y_{ij}$ (mm/yr), is estimated based on annual average precipitation and the Budyko curve (Budyko 1974) as follows:

$$Y_{ij}=\left(1-\frac{AET_{ij}}{P_{i}}\right)\times P_{i}$$

where, $AET_{ij}$ (mm/yr) is the annual actual evapotranspiration for pixel $i$ on $LULC_{j}$ , and$P_{i}$ (mm/yr) is the annual precipitation for pixel $i$ .

For vegetated LULC, the evapotranspiration portion of the water balance, $\frac{AET_{ij}}{P_{i}}$, is based on an expression of the Budyko curve proposed by Fu 1981 and Zhang et al. 2004:

$$\frac{AET_{ij}}{P_{i}}=1+\frac{PET_{ij}}{P_{i}}-\left[1+\left(\frac{PET_{ij}}{P_{i}}\right)^{\omega_{i}}\right]^{\frac{1}{\omega_{i}}}$$

where $PET_{ij}$ is the potential evapotranspiration and $\omega_{i}$ is a non-physical parameter that characterizes the natural climatic-soil properties.

Potential evapotranspiration $PET_{ij}$is defined as:

$$PET_{ij}=k_{ij}\times ET_{0_{i}}$$

where $ET_{0_{i}}$is the reference evapotranspiration from pixel $i$ and $k_{ij}$is the vegetation evapotranspiration coefficient associated for pixel $i$ on $LULC_{j}$.

$$\omega_{i}=Z\cdot\frac{AWC_{i}}{P_{i}}+1.25$$

$$AWC_{i}=min\left(rest\_layer\_depth, root\_depth\right)\times PAWC$$

where $\omega_{i}$ is a non-physical parameter that characterizes the natural climatic-soil properties; $Z$ is a dimensionless constant that represents the local features of precipitation and hydrogeology, ranging from 1 to 30; $AWC_{i}$ (mm) is the volumetric plant available water content; the *1.25* term is the minimum value of $\omega_{i}$ (Donohue et al. 2012); $PAWC$ (mm) is the plant available water capacity.

For non-vegetated LULC (e.g., open water, developed land), the annual actual evapotranspiration is computed directly from the reference evapotranspiration and has an upper limit defined by the precipitation:

$$AET_{ij}=min\left(k_{ij}\times ET_{0_{i}}, P_{i}\right)$$

where $k_{ij}$ is evapotranspiration coefficient for pixel $i$ on $LULC_{j}$, $ET_{0_{i}}$is the reference evapotranspiration from pixel $i$and $P_{i}$ (mm/yr) is the annual precipitation for pixel $i$.

In the current study, we define “water scarcity” as the discrepancy between the total water yield and the total demand of water, to measure the satisfaction of water retention service. Water scarcity is calculated using the *Realized Supply model*:

$$W_{s}=Y_{t}-D_{t}$$

where $W_{s}$(m^3^/yr) is the estimated water scarcity, $Y_{t}$(m^3^/yr) is the total water yield, $D_{t}$(m^3^/yr) is the total volume of water demand including the industrial, agricultural, domestic and ecological components.

**Nutrient Delivery Ratio (NDR) model:** Although there are multiple potential factors affecting water quality, the main causes of water pollution in the THB are nitrogen and phosphorus pollutants, as indicated by the frequent outbreaks of cyanobacteria (Guo 2007; Qin et al. 2010). Therefore, the water purification service in this study refers to the ecosystem’s capacity to absorb nitrogen and phosphorus from the water flows. Sources of nutrient across the landscape are determined based on the land use map and associated loading rates. Each pixel’s actual load is modified based on the precipitation to account for the local runoff potential. Nutrient export from each pixel is calculated based on the product of the load and the nutrient delivery ratio.

The NDR model illustrates the process of nutrients export to streams, and the exported nutrients are calculated as follows:

$$\left\{\begin{matrix} X_{\exp ort_{i}}=load_{surf,i}\times NDR_{surf,i}+load_{subs,i}\times NDR_{subs,i} \\ X_{\exp ort_{tot}}=\sum_{i} X_{\exp ort_{i}} \end{matrix}\right.$$

Based on the original load, each pixel’s estimated load is modified by the local runoff potential index, and the ratio between surface and subsurface nutrient sources is given by the parameter$proportion\_subsurface_{i}$. Therefore, the load (kg·ha^-1^·yr^-1^) for pixel $i$ is given by:

$$\left\{\begin{matrix} load_{surf,i}=(1-proportion\_subsurface_{i})\times modified\_load_{i} \\ load_{subsurf,i}=proportion\_subsurface_{i})\times modified\_load_{i} \end{matrix}\right.$$

$$\left\{\begin{matrix} modified\_load_{i}=load_{i}\times RPI_{i} \\ RPI_{i}=\frac{RP_{i}}{RP_{a}} \end{matrix}\right.$$

where $RPI_{i}$ is the runoff potential index for pixel $i$, $RP_{i}$is the nutrient runoff proxy for runoff on pixel $i$ and $RP_{a}$ is the average $RP$ over the raster.

Precisely, the delivery ratios $(NDR_{surf,i}, NDR_{subs,i})$ are calculated as follows:

(1) Surface NDR: The surface NDR is determined by the ability of downstream pixels to transport nutrient,$NDR_{0,i}$, together with the topographic index, $IC$, for pixel $i$:

$$NDR_{surf,i}=NDR_{0,i}\times\left(1+\exp\left(\frac{IC_{i}-IC_{0}}{k}\right)\right)^{-1}$$

where $IC_{0}$ and $k$ are calibration parameters; $IC_{i}$ is a topographic index, and $NDR_{0,i}$is the proportion of nutrient that is not retained by downstream pixels.

$$NDR_{0,i}=1-eff_{i}^{'}$$

$$eff_{i}^{'}=\left\{\begin{matrix} eff_{LULC_{j}}\times\left(1-s_{i}\right) if down_{i} is a stream pixel \\ eff_{down_{i}}^{'}\times s_{i}+eff_{LULC_{j}}\times\left(1-s_{i}\right) if eff_{LULC_{j}}>eff_{down_{i}}^{'} \\ eff_{down_{i}}^{'} otherwise \end{matrix}\right.$$

where $eff_{i}^{'}$ is retention efficiency for pixel $i$, $eff_{LULC_{j}}$is the maximum retention efficiency of $LULC_{j}$, $eff_{down_{i}}^{'}$is the retention efficiency on the pixel directly downstream from pixel $i$, and $s_{i}$is the step factor defined as:

$$s_{i}=\exp\left(\frac{-5l_{i_{down}}}{l_{LULC_{i}}}\right)$$

where $l_{i_{down}}$ is the length of the flow path from pixel $i$ to its downstream neighbour, $l_{LULC_{i}}$ is the LULC retention length on pixel $i$ .

$IC$ is the index of connectivity:

$$IC=log_{10}\left(\frac{D_{up}}{D_{dn}}\right)$$

$$D_{up}=\overline{s}\sqrt{A}, D_{dn}=\sum_{i} \frac{d_{i}}{s_{i}}$$

where $\overline{s}$(m/m) and $A$ (m^2^) are the average slope gradient and the total area of the upslope contributing area, respectively, $d_{i}$ (m) is the length of the flow path along the pixel $i$.

(2) Subsurface NDR:

$$NDR_{subs,i}=1-eff_{subs}\times\left(1-e^{\frac{-5l_{i}}{l_{subs}}}\right)$$

where $eff_{subs}$is the maximum nutrient retention efficiency the subsurface flow can reach, $l_{i}$ is the distance from the pixel to the stream, $l_{subs}$ is the subsurface flow retention length.

**Sediment Delivery Ratio (SDR) model:** Soil retention service accounts for the retention by vegetation of rainfall-eroded soil, for the protection of soil resources and water quality. The InVEST sediment delivery model is to map overland sediment generation and delivery to the stream. The sediment export from a pixel $i$, $Export_{i}$ (ton·ha^-1^·yr^-1^), and the total sediment export of the evaluate area, $Export_{tot}$ (ton·ha^-1^·yr^-1^), is given by:

$$\left\{\begin{matrix} Export_{i}=usle_{i}\times SDR_{i} \\ Export_{tot}=\sum_{i} Export_{i} \end{matrix}\right.$$

The amount of annual soil loss on pixel $i$, $usle_{i}$, is given by the revised universal soil loss equation:

$$usle_{i}=R_{i}\times K_{i}\times LS_{i}\times C_{i}\times P_{i}$$

where $R_{i}$ (MJ·mm·(ha·hr)^-1^) is the rainfall erosivity, $K_{i}$ (ton·ha·hr·(MJ·ha·mm)^-1^) is the soil erodibility, $LS_{i}$ is the slope length-gradient factor, $C_{i}$ is the crop-management factor, and $P_{i}$ is the support practice factor.

The connectivity index $IC$ is given by:

$$IC=log_{10}\left(\frac{D_{up}}{D_{dn}}\right)$$

$D_{up}$ is the upslope component defined as:

$$D_{up}=\overline{C}\times\overline{S}\times\sqrt{A}$$

where $\overline{C}$ is the average $C$ factor of the upslope contributing area, $\overline{S}$ (m/m) is the average slope gradient of the upslope contributing area and $A$ (m^2^) is the upslope contributing area, and, $D_{dn}$ is the downslope component, defined as:

$$D_{dn}=\sum_{i} \frac{d_{i}}{C_{i}S_{i}}$$

where $C_{i}$ and $S_{i}$ are the $C$ factor and the slope gradient on pixel $i$, $d_{i}$ (m) is the length of the flow path along the pixel $i$.

The SDR for a pixel $i$, $SDR_{i}$, is derived from the connectivity index $IC$:

$$SDR_{i}=\frac{SDR_{max}}{1+\exp\left(\frac{IC_{0}-IC_{i}}{k}\right)}$$

where $SDR_{max}$ is the maximum theoretical SDR, $IC_{0}$ and $k$ are calibration parameters that define the shape of the SDR-IC relationship.
