# Supplementary figures and images for: Spatio-temporal evolution of water-related ecosystem services: Taihu Basin, China
Source: PeerJ. 2018 Jun 22;6:e5041. doi: 10.7717/peerj.5041 (PMC6016528; doi:10.7717/peerj.5041)

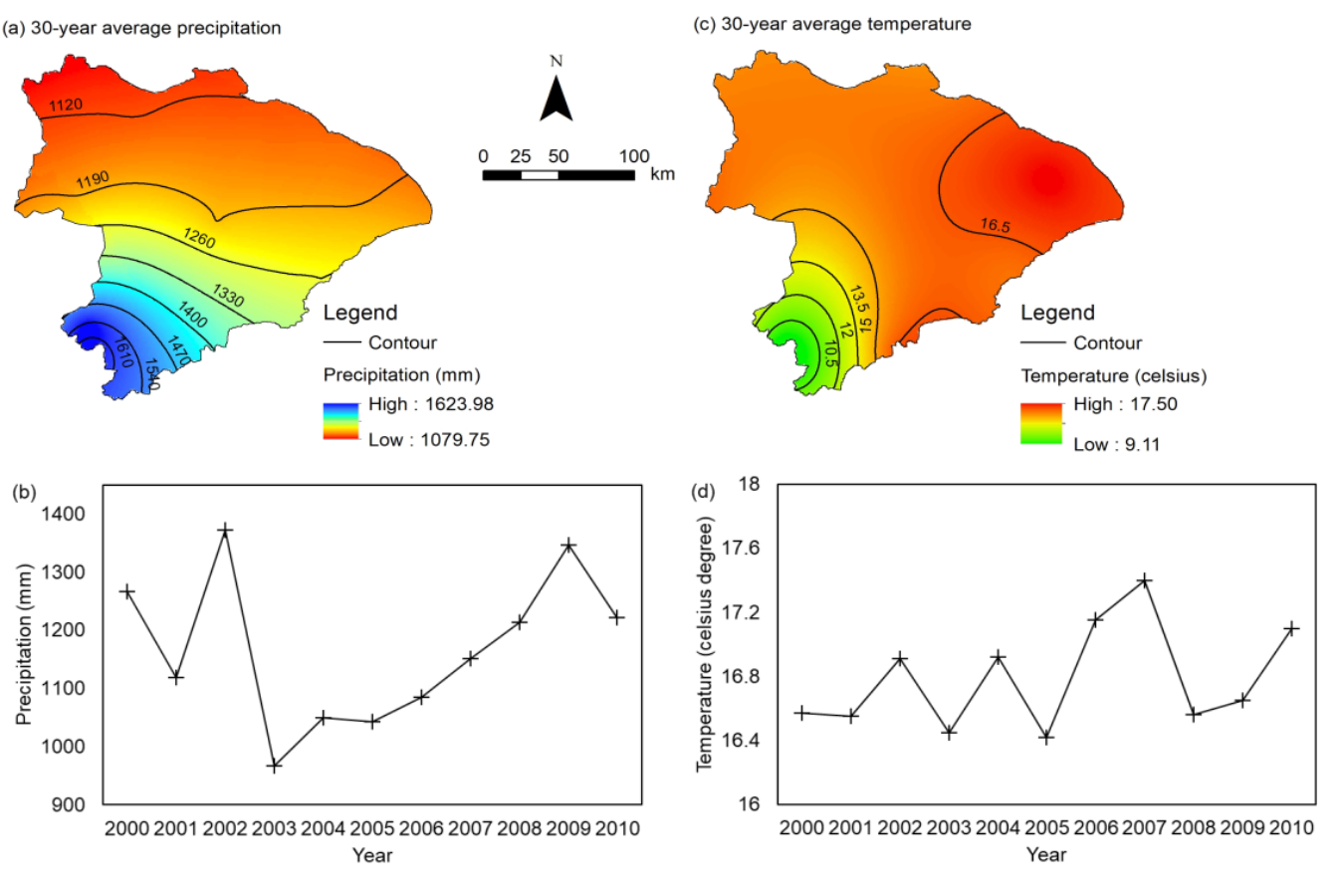

Supplement: Supplemental Information 2 — Four sub-figures indicate the spatio-temporal condition of precipitation and temperature in the Taihu Basin. [file peerj-06-5041-s002.png]
