# Supplementary material for: Spatio-temporal evolution of water-related ecosystem services: Taihu Basin, China
Source: PeerJ. 2018 Jun 22;6:e5041. doi: 10.7717/peerj.5041 (PMC6016528; doi:10.7717/peerj.5041)
Supplement: Supplemental Information 3 — This table lists collected spatial data and other relevant crucial data for the Taihu Basin water-related ecosystem services evaluation. It also summarizes each dataset by their sources, and includes a short introduction and the associated models. [file peerj-06-5041-s003.docx]

**Supplemental file 3 (SF 3):**

**Table S1** Data requirement for the InVEST model (Water yield model=WY; Nutrient delivery ratio model=NDR; Sediment delivery ratio model=SDR)

| **Data** | **Type** | **Data source** | **Note** | **Related model** |
| --- | --- | --- | --- | --- |
| Digital Elevation Model (DEM) | Raster | Geospatial Data Cloud, http://www.gscloud.cn | Resolution is 30m×30m | NDR, SDR |
| Annual average precipitation | Raster | China Meteorological Data Center, http://data.cma.cn/ | Interpolated based on annual data,  resolution is 30m×30m | WY, NDR, SDR |
| Reference evapotranspiration | Raster | MODIS Global Evapotranspiration Project (MOD16)  http://www.ntsg.umt.edu/project/mod16 | Resolution is 30m×30m | WY |
| Plant available water content | Raster | Environmental and Ecological Science Data Center for West China, http://westdc.westgis.ac.cn/ | Calculated based on the soil data (Harmonized World Soil Database) according to the model proposed by Zhou (2005), resolution is 30m×30m | WY |
| Land use / land cover | Raster | Resource and environment data cloud platform,  Chinses Academy of Sciences, http://www.resdc.cn/ | LULC of year 2000, 2005 and 2010, including forest, grassland, open water, garden, urban greenland, developed land, cultivated land and bare land, resolution is 30m×30m | WY, NDR, SDR |
| Depth to root restricting layer | Raster | Environmental and Ecological Science Data Center for West China, http://westdc.westgis.ac.cn/ | Derived from the soil data (Harmonized World Soil Database), resolution is 30m×30m | WY |
| Watersheds | Shapefile | Geospatial Data Cloud, http://www.gscloud.cn | A shapefile determined by DEM raster using ArcGIS tool | WY, NDR, SDR |
| Rainfall erosivity index | Raster | China Meteorological Data Center, http://data.cma.cn/ | Calculated based on precipitation according to the model proposed by Zhang and Fu (2003), resolution is 30m×30m | SDR |
| Soil erodibility | Raster | China Meteorological Data Center, http://data.cma.cn/ | Calculated based on precipitation according to the model proposed by Cao et al. (2015), resolution is 30m×30m | SDR |
| Biophysical table | .CSV file | Literatures ( Wang et al. 2016; Han et al. 2016), and the InVEST user’s guide (Sharp et al. 2016) | Including attributes of each LULC, K_c_ (the plant evapotranspiration coefficient), load of nutrients, efficiency of nutrient retention, etc. | WY, NDR, SDR |
| Water demand table | .CSV file | Taihu Basin & Southeast Rivers Water Resources Bulletin (2000, 2005, 2010), www.tba.gov.cn | Computed based on the annual demand of water resources in THB | WY |
