# Supplementary material for: Spatio-temporal evolution of water-related ecosystem services: Taihu Basin, China
Source: PeerJ. 2018 Jun 22;6:e5041. doi: 10.7717/peerj.5041 (PMC6016528; doi:10.7717/peerj.5041)
Supplement: Supplemental Information 4 — This table lists the key parameters required by the InVEST model. [file peerj-06-5041-s004.docx]

**Supplemental file 4 (SF 4):**

**Table S2** Key parameters used in the current study

| Parameters | Description | Computation |
| --- | --- | --- |
| $\text{ω}_{\text{i}}$ | A non-physical parameter that characterizes the natural climatic-soil properties | $\text{ω}_{\text{i}}\text{=Z}\frac{\text{AWC}_{\text{i}}}{\text{P}_{\text{i}}}\text{+1.25}$, $\text{Z}$ is an seasonality constant that captures the local precipitation pattern and hydrogeological characteristics, ranging from 1 to 30;$\text{AWC}_{\text{i}}$ (mm) is the volumetric plant available water content; the 1.25 term is the minimum value of $\text{ω}_{\text{i}}$ ; $\text{P}_{\text{i}}$ (mm/yr) is the annual precipitation for pixel $\text{i}$ |
| $\text{Z}$ | An empirical constant that captures the local precipitation pattern and hydrogeological characteristics | Estimated as $\text{0.2×N}$, $\text{N}$ where is the average number of rain days (>1mm) per year over the study period (Donohue et al., 2012) |
| $\text{AWC}_{\text{i}}$ | The volumetric plant available water content | $\text{AWC}_{\text{i}}\text{=min(}\text{rest}\text{\_}\text{layer}\text{\_}\text{depth}\text{, root}\text{\_}\text{depth)×PAWC}$ , $\text{PAWC}$ (mm) is the plant available water capacity; root restricting layer depth is the soil depth at which root penetration is inhibited because of physical or chemical characteristics; vegetation rooting depth is given as the depth at which 95% of a vegetation type’s root biomass occurs |
| $\text{k}_{\text{ij}}$ | Evapotranspiration coefficient for each pixel | Defined according to literatures (Wang et al. 2016) and the InVEST user’s guide (Sharp et al. 2016) |
| $\text{RPI}_{\text{i}}$ | The runoff potential index for each pixel | $\text{RPI}_{\text{i}}\text{=}\frac{\text{RP}_{\text{i}}}{\text{RP}_{\text{a}}}$ , $\text{RP}_{\text{i}}$ is the nutrient runoff proxy for runoff on pixel $\text{i}$ , and $\text{RP}_{\text{a}}$ is the average $\text{RP}$ over the raster |
| $\text{eff\_n}$  $\text{eff\_p}$ | The maximum retention efficiency of nitrogen and phosphorus for each LULC, varying between 0 and 1. | Defined according to the literature data and the InVEST user’s guide (Sharp et al. 2016) |
| $\text{IC}$ for NDR model | The index of connectivity | $\text{IC=}\text{log}_{\text{10}} \text{( }\frac{\text{D}_{\text{up}}}{\text{D}_{\text{dn}}}\text{ )}$, $\text{D}_{\text{up}}\text{=}\bar{\text{S}}\sqrt{\text{A}}\text{, D}_{\text{dn}}\text{=}\sum_{\text{i}} \frac{\text{d}_{\text{i}}}{\text{S}_{\text{i}}}$ , $\bar{\text{S}}$ (m/m) is the average slope gradient of the upslope contributing area and $\text{A}$ (m^2^) is the upslope contributing area, $\text{d}_{\text{i}}$ (m) is the length of the flow path along the pixel $\text{i}$ |
| $\text{usle}_{\text{i}}$ | The amount of annual soil loss on each pixel | $\text{usle}_{\text{i}}\text{=}\text{R}_{\text{i}}\text{×}\text{K}_{\text{i}}\text{×}\text{LS}_{\text{i}}\text{×}\text{C}_{\text{i}}\text{×}\text{P}_{\text{i}}$ , where, $\text{R}_{\text{i}}$(MJ·mm·(ha·hr)^-1^) is the rainfall erosivity, $\text{K}_{\text{i}}$(ton·ha·hr·(MJ·ha·mm)^-1^) is the soil erodibility, $\text{LS}_{\text{i}}$ is the slope length-gradient factor, $\text{C}_{\text{i}}$ is the crop-management factor, and $\text{P}_{\text{i}}$ is the support practice factor |
| $\text{IC}$ for SDR model | The index of connectivity | $\text{IC=}\text{log}_{\text{10}} \text{( }\frac{\text{D}_{\text{up}}}{\text{D}_{\text{dn}}}\text{ )}$ , $\text{D}_{\text{up}}$ is the upslope component defined as: $\text{D}_{\text{up}}\text{=}\bar{\text{C}}\bar{\text{S}}\sqrt{\text{A}}$ , where, $\bar{\text{C}}$ is the average $\text{C}$ factor of the upslope contributing area, $\bar{\text{S}}$(m/m) is the average slope gradient of the upslope contributing area and $\text{A}$ (m^2^) is the upslope contributing area  and, $\text{D}_{\text{dn}}$ is the downslope component defined as: $\text{D}_{\text{dn}}\text{=}\sum_{\text{i}} \frac{\text{d}_{\text{i}}}{\text{C}_{\text{i}}\text{S}_{\text{i}}}$ , where $\text{C}_{\text{i}}$ and $\text{S}_{\text{i}}$ are the $\text{C}$ factor and the slope gradient on pixel $\text{i}$, $\text{d}_{\text{i}}$(m) is the length of the flow path along the pixel $\text{i}$ |
| $\text{SDR}_{\text{max}}$ | The maximum theoretical *SDR* | Defined as 0.8 according to the InVEST user’s guide |
