# Supplementary material for: Spatio-temporal evolution of water-related ecosystem services: Taihu Basin, China
Source: PeerJ. 2018 Jun 22;6:e5041. doi: 10.7717/peerj.5041 (PMC6016528; doi:10.7717/peerj.5041)
Supplement: Supplemental Information 5 — This is the required biophysical table for the InVEST model. [file peerj-06-5041-s005.docx]

**Supplemental file 5 (SF 5):**

**Table S3** Biophysical table (Wang et al. 2016, Sharp et al. 2016, China Soil Map Based on Harmonized World Soil Database 2015). Kc: the plant evapotranspiration coefficient for each LULC classe; root_depth: the maximum root depth for vegetated land use classes in mm ; usle_c and usle_p: two parameters in the SDR model (equation 19); sedret_eff: the maximum soil retention efficiency for each LULC ; load_n: the original nitrogen loading for each LULC in kg/ha•yr; load_p: the original phosphorus loading for each LULC in kg/ha•yr; eff_n: the maximum nitrogen retention efficiency for each LULC; eff_p: the maximum phosphorus retention efficiency for each LULC; LULC_vegetation: a flag to distinguish barren land with vegetated land, 1 for vegetated land and 0 for others.

| LULC_description | LULC_code | Kc | root_depth (mm) | usle_c | usle_p | sedret_eff | load_n | eff_n | load_p | eff_p | LULC_vegetation |
| --- | --- | --- | --- | --- | --- | --- | --- | --- | --- | --- | --- |
| Forest | 1 | 1 | 7000 | 0.003 | 0.2 | 0.6 | 8 | 0.8 | 1.6 | 0.8 | 1 |
| Grassland | 2 | 0.8 | 2000 | 0.01 | 0.2 | 0.4 | 10 | 0.4 | 3 | 0.4 | 1 |
| Water | 3 | 1 | 1000 | 0.001 | 0.001 | 0.8 | 0.001 | 0.05 | 0.001 | 0.05 | 0 |
| Garden | 4 | 0.6 | 3000 | 0.006 | 0.3 | 0.3 | 20 | 0.45 | 6 | 0.45 | 1 |
| Developed | 5 | 0.3 | 500 | 0.001 | 0.001 | 0 | 30 | 0 | 8 | 0 | 0 |
| Urban green | 6 | 0.5 | 2000 | 0.01 | 0.2 | 0.5 | 6 | 0.5 | 0.06 | 0.5 | 1 |
| Cultivated | 7 | 0.6 | 1000 | 0.35 | 0.4 | 0.25 | 80 | 0.2 | 24 | 0.2 | 1 |
| Bared | 8 | 0.2 | 500 | 0.01 | 0.2 | 0.05 | 10 | 0.05 | 0.14 | 0.05 | 0 |
